# Efficacy and safety of nab-paclitaxel in advanced or recurrent small cell lung cancer: a single-arm meta-analysis

**DOI:** 10.3389/fphar.2026.1852047

**Published:** 2026-06-11

**Authors:** Jun-Chen Liu, Hong-Jing Yu, Zhi Chen, Qi Deng

**Affiliations:** 1 Department of Clinical Pharmacy, The First People’s Hospital of Jiande, Jiande, China; 2 Department of Medical Oncology, The First People’s Hospital of Jiande, Zhejiang, China

**Keywords:** advanced/recurrent small cell lung cancer, adverse events, albumin-bound paclitaxel, disease control rate, objective response rate

## Abstract

**Background:**

Although various effective agents used as second- and third-line treatments for advanced or recurrent small cell lung cancer have improved overall survival, the optimal therapeutic regimen remains controversial. Previous studies have shown that albumin-bound paclitaxel exhibits favorable anticancer activity in many cancer types. However, almost all studies of albumin-bound paclitaxel for recurrent small cell lung cancer are non-randomized controlled trials with small sample sizes, diverse first-line treatment regimens, and non-controlled data analyses. This may result in a lack of valid indicators for evaluating the efficacy and safety of albumin-bound paclitaxel. Therefore, this meta-analysis aims to assess the efficacy and safety of albumin-bound paclitaxel in patients with advanced or recurrent small cell lung cancer.

**Methods:**

PubMed, Embase, the Cochrane Library, and Web of Science databases were systematically searched for relevant studies. Outcomes including overall response rate (ORR), disease control rate (DCR), progression-free survival (PFS), overall survival (OS), and adverse events (AEs) were extracted for further analysis.

**Results:**

Nine studies involving 334 patients were enrolled in this meta-analysis. In terms of tumor response, the pooled ORR and DCR were 25.0% and 62.5%, respectively. With regard to survival analysis, the pooled PFS and OS were 3.16 months and 7.23 months, respectively. The most common treatment-related adverse events of nab-paclitaxel were Leukopenia (all grade: 59.3%, ≥grade III: 14.1%), Neutropenia (all grade: 54.9%, ≥grade III: 14.7%), and Anemia (all grade: 53.0%, ≥grade III: 6.2%). Subgroup analysis revealed that combination therapy with immunotherapy was associated with numerically higher response rates and survival.

**Conclusions:**

In summary, this meta-analysis demonstrated that albumin-bound paclitaxel has moderate efficacy and manageable safety in the later-line treatment of advanced or recurrent small cell lung cancer.

**Systematic Review Registration:**

https://www.crd.york.ac.uk/PROSPERO/view/CRD420261350933, identifier 420261350933.

## Introduction

Lung cancer is the leading cause of cancer-related mortality worldwide. Small cell lung cancer (SCLC) accounts for approximately 15% of all lung cancer cases (Gazdar et al., 2017). As a high-grade pulmonary neuroendocrine tumor and a highly malignant subtype of lung cancer, SCLC is characterized by rapid growth and extensive metastasis. More than 60% of patients present with stage IV disease at diagnosis (Nicholson et al., 2016). The high recurrence rate after first-line treatment poses a major clinical challenge (Zugazagoitia and Paz-Ares, 2022). Currently, etoposide plus cisplatin or carboplatin (EP) combined with immunotherapy has become the standard first-line regimen for extensive-stage small cell lung cancer (ES-SCLC) (Cheng et al., 2022; Horn et al., 2018; Paz-Ares et al., 2019; Wang et al., 2022). However, the efficacy of these therapeutic strategies remains limited, and patients frequently experience disease progression or recurrence within a short period. Therefore, there is an urgent need to explore effective second-line treatment strategies. During disease progression, the efficacy of subsequent therapies is affected by the response to first-line chemotherapy, resulting in low overall response rates and limited available therapeutic agents. Recommended chemotherapeutic agents for relapsed small cell lung cancer (SCLC) in clinical guidelines include topotecan, irinotecan, temozolomide, paclitaxel, gemcitabine, and docetaxel (Eckardt et al., 2007; Masters et al., 2003; Pietanza et al., 2012). According to the 2026 National Comprehensive Cancer Network (NCCN) Guidelines for Small Cell Lung Cancer (Version 2.2026) (Ganti et al., 2026), second-line treatment options for relapsed/refractory SCLC mainly comprise taratumumab, irinotecan, lurbinectedin, topotecan,and immune checkpoint inhibitors (ICIs). As a conventional standard second-line agent, topotecan yields an objective response rate (ORR) of only 10%-15%, accompanied by severe toxicities such as myelosuppression (Peters et al., 2024). Although lurbinectedin is recommended as a preferred option for first-line maintenance and second-line therapy in updated guidelines, its ORR is merely approximately 35%, with further diminished efficacy in heavily pretreated patients; moreover, 30.5% of patients may discontinue treatment due to adverse events. As monotherapy for relapsed/refractory SCLC, ICIs achieve an ORR of less than 15% and benefit only a subset of patients (Forde et al., 2022; Schmid et al., 2020). The occurrence of lung cancer is intricately associated with the host immune surveillance status. Epidemiological evidence has demonstrated that individuals with allergic diseases such as allergic rhinitis may have a lower risk of developing lung cancer (Yang et al., 2025). Nevertheless, once tumors develop and progress to advanced small cell lung cancer (SCLC), the profoundly immunosuppressive tumor microenvironment frequently leads to the failure of conventional therapies. Therefore, it is particularly urgent to explore therapeutic strategies—such as chemotherapy combined with immunotherapy—that can effectively reverse this immune suppression and improve the prognosis of patients with recurrent disease.

Solvent-based paclitaxel also demonstrates antitumor activity in refractory relapsed SCLC (Smit et al., 1998), but carries a substantial risk of severe hypersensitivity reactions. In contrast to solvent-based paclitaxel, nanoparticle albumin-bound paclitaxel (nab-PTX) is a novel solvent-free paclitaxel formulation that achieves targeted delivery of paclitaxel to tumor tissues via an albumin nanoparticle carrier. It does not require premedication with glucocorticoids for hypersensitivity prophylaxis, and can significantly increase intratumoral drug concentration, improve bioavailability, shorten infusion duration, and reduce systemic toxicities (Yardley, 2013; Desai et al., 2006; Ibrahim et al., 2002). In several retrospective studies (Ikeda et al., 2022; Li et al., 2024; Mu et al., 2023; Wan et al., 2023), nab-paclitaxel has shown anticancer activity in patients with relapsed SCLC. However, most current studies of nab-PTX for advanced or relapsed SCLC are single-center, small-sample single-arm trials. Variations in baseline patient characteristics and treatment regimens (dosage, cycles) across studies lead to considerable heterogeneity in efficacy and safety outcomes. Reported objective response rates range from 5.6% to 40.7%, and median progression-free survival ranges from 1.8 to 5.0 months, making it difficult to comprehensively and accurately reflect the true therapeutic value of nab-PTX in this patient population. As an important evidence-based medical approach, single-arm meta-analysis enables systematic review and integration of data from all relevant single-arm studies, quantitatively evaluates the efficacy and safety of interventions, reduces bias from individual small-sample studies, and provides high-quality evidence for clinical treatment decisions (Begg and Pilote, 1991).

To date, there has been no single-arm meta-analysis specifically focusing on nab-PTX for advanced or relapsed SCLC. This gap prevents the systematic integration of existing research evidence and makes it difficult to clarify the optimal population, therapeutic dosage, and toxicity management strategies for nab-PTX. Therefore, this study intends to adopt a single-arm meta-analysis approach to systematically search for published single-arm studies worldwide on nab-PTX in the treatment of advanced or relapsed SCLC. We will integrate data on its efficacy (objective response rate, progression-free survival, overall survival, disease control rate) and safety to clarify the overall therapeutic effect and safety profile of nab-PTX in this patient population. This study aims to provide reliable evidence-based support for clinical treatment decisions and offer a reference for the conduct of large-scale randomized controlled trials in the future.

## Materials and methods

### Search strategy

Two reviewers independently performed a comprehensive systematic literature search. PubMed, Embase, Cochrane Library, and Web of Science were searched for relevant studies published up to January 7, 2026. The major search terms that were used were (“small Cell Lung Cancer” or “SCLC” or “small Cell Lung Neoplasm” or “Lung Neoplasm” or “Lung Cancer”) and (“nabpaclitaxel” or “nab-PTX” or “albumin-PTX” or “albumin-Bound paclitaxel” or “Abraxane”) AND “therapy” and “efficacy”. Any disagreements between the two investigators were resolved by discussion until consensus was reached.

### Selection criteria

Studies eligible for this meta-analysis were required to meet the following predefined criteria: 1) Population: patients with pathologically confirmed advanced or recurrent small cell lung cancer (SCLC), regardless of histological subtype; 2) Intervention: nanoparticle albumin-bound paclitaxel (nab-paclitaxel) administered as monotherapy or combined with chemotherapy; 3) Study design: phase II clinical trials or retrospective observational studies; 4) Outcomes: at least one key endpoint reported, including objective response rate (ORR), disease control rate (DCR), progression-free survival (PFS), overall survival (OS), or adverse events (AEs). Tumor response was evaluated per RECIST version 1.1 (Eisenhauer et al., 2009), and toxicity was graded using CTCAE (Trotti et al., 2003).

Studies were excluded if they were non-clinical studies, reviews, meta-analyses, case reports, letters, or duplicate publications; if the sample size was fewer than 15 patients; or if patients had not received prior first-line chemotherapy. Two independent reviewers screened all candidate studies according to the above criteria.

### Data extraction and quality assessment

Two researchers independently extracted the core data from all eligible studies, and methodological quality evaluation of the included literature was conducted subsequently. The key characteristics extracted from each study were summarized as follows: authors, publication year, research design, sample size, treatment regimen, median age, and documented clinical endpoints. Clinical efficacy and safety indicators included objective response rate (ORR), disease control rate (DCR), overall survival (OS), progression-free survival (PFS), overall incidence of adverse events (AEs), and the proportion of grade ≥3 adverse events. To ensure data reliability, the above data extraction and verification work was completed independently by the two investigators separately.

Methodological quality of the included non-comparative studies was evaluated using the Newcastle–Ottawa Scale (NOS) (Stang, 2010). In addition, all retrospective case series were critically appraised employing the JBI Critical Appraisal Checklist for Case Series (Tjb and Institute, 2016).

### Statistical analysis

All statistical analyses in this meta-analysis were conducted using STATA 18.0 software (StataCorp LP, College Station, TX, USA). Study heterogeneity was quantified by the chi-squared test and I^2^ statistic, with a p-value < 0.1 considered indicative of significant heterogeneity. A random-effects model was applied in the presence of significant heterogeneity (p < 0.1 and I^2^ > 50%); otherwise, a fixed-effects model was adopted (Yao et al., 2021). Moreover, sensitivity analysis was performed to analyze the stability and reliability of the pooled results.

## Result

### Study selection

**FIGURE 1:**
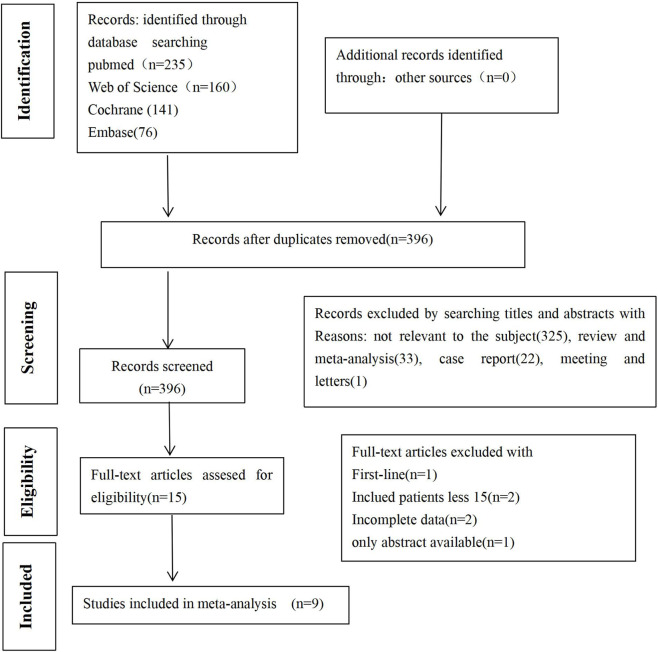
Flow diagram of meta-analysis for inclusion/exclusion of studies.

A total of 612 records were initially identified from the four databases (PubMed = 235, Embase = 76, Web of Science = 160, and Cochrane Library = 141). A total of 15 studies were retained after excluding duplicates and initial screening of titles and abstracts. Subsequently, the remaining full-text articles were carefully assessed. Six studies were excluded due to first-line treatment setting, small sample size, or incomplete data. Finally, 9 studies with a total of 334 patients met the inclusion criteria and were included in this meta-analysis (Ikeda et al., 2022; Li et al., 2024; Mu et al., 2023; Wan et al., 2023; Byrne et al., 2024; Gelsomino et al., 2020; Nakao et al., 2020; Oi et al., 2022; Wang et al., 2021). The flowchart illustrating the study selection process is presented in Figure 1, and the detailed information of each included study is summarized in Table 1.

**TABLE 1:** Characteristics of the included comparative studies.

Study, year	Study design	Treatment arms	Number	Median age	Endpionts
Xiaobing LI 2024	Retrospective	200 mg/m^2^ nab-P + 12 mg anlotinib + Anti-PD-1, every 3 weeks	45	68	ORR, DCR, PFS, OS, and AEs
Ikeda, N 2022	Phase II	100 mg/m^2^ nab-P weekly + C (under the curve = 5), every 3 weeks	21	70	ORR, DCR, PFS, OS, and AEs
Rui wan 2023(A)	Retrospective	130 mg/m^2^ nab-P, every 3 weeks	29	57	ORR, DCR, PFS, OS, and AEs
Rui wan 2023(B)	Retrospective	130 mg/m^2^ nab-P+Anti-PD-1, every 3 weeks	27	59	ORR, DCR, PFS, OS, and AEs
Fengchun mu 2023	Retrospective	130 mg/m^2^ nab-P+Anti-PD-1, every 3 weeks	40	NR	ORR, DCR, PFS, OS, and AEs
Nakao M 2020	Retrospective	nab-P + Anti-PD-1/PD-L1	17	71	ORR, DCR, PFS, OS, and AEs
Yuchao wang 2021	Retrospective	75-100 mg/m^2^ nab-P weekly, every 4 weeks	37	62	ORR, DCR, PFS, OS, and AEs
Byrne, M.M. 2024	Phase II	130 mg/m^2^ nab-P, every 3 weeks	32	65	ORR, DCR, PFS, OS, and AEs
Oi H 2022	Retrospective	100 mg/m^2^ nab-P + GEM1g/m^2^, every 3 weeks	18	73	ORR, DCR, PFS, OS, and AEs
Gelsomino, F 2020	Phase II	80 mg/m^2^ nab-P weekly, every 4 weeks	68	69	ORR, DCR, PFS, OS, and AEs

NR, not reported; nab-P: nab-paclitaxel; C: carboplatin; GEM:gemcitabine; ORR: overall response rate; PFS: progression-free survival; OS: overall survival; DCR, disease control rate; AEs, adverse events.

### Quality assessment

Four non-randomized studies were quality-evaluated using the Newcastle–Ottawa Scale (NOS). This tool scores studies across eight items covering three core domains: participant selection, comparability between groups, and assessment of outcomes (for cohort studies) or exposure status (for case–control studies). Five retrospective case series were appraised using the JBI Critical Appraisal Checklist for Case Series (Stang, 2010), a ten-item instrument that evaluates methodological quality with respect to case selection, diagnostic ascertainment, and detailed presentation of clinical data (Munn et al., 2020). The details of the quality assessment are presented in Table 2.

**TABLE 2:** Quality assessment of the studies included in the meta-analysis.

JBI critical appraisal checklist for case series for included retrospective studies												
Study	Year	Q1	Q2	Q3	Q4	Q5	Q6	Q7	Q8	Q9	Q10	Total score
Xiaobing LI	2024	1	0	1	1	1	1	0	1	1	1	8
Ikeda, N	2022	1	0	1	1	1	1	1	1	1	1	9
Nakao M	2020	1	1	1	1	1	1	0	1	1	1	9
Yuchao Wang	2021	1	1	1	1	1	1	1	1	1	1	10
Byrne, M.M.	2024	1	1	1	1	1	1	0	1	1	1	9

Newcastle–Ottawa scale (NOS) for included non-randomized studies												
Study	Year	I	II	III	IV	V	VI	VII	VIII	Total score	​	
Rui Wan	2022	1	1	1	1	1	1	1	1	8	​	
Fengchun Mu	2023	1	1	1	1	1	1	1	1	8		
Gelsomino, F	2020	1	1	1	1	1	1	1	1	8		
Oi H	2022	1	1	1	1	0	1	1	1	7		

JBI, Critical Appraisal Checklist for Case Series (Q1-Q10). Scoring Rule: Yes = 1 point, No/Unclear/Not applicable = 0 point; Total score = 10 points. Q1, Were clear inclusion and exclusion criteria reported for the study population? Q2, Were participants recruited consecutively? Q3, Were baseline demographic and clinical characteristics of all participants clearly described? Q4, Were the study setting and study period clearly stated? Q5, Were study outcomes assessed using objective and standardized methods? Q6, Was the follow-up period sufficient and clearly reported? Q7: Were all dropouts or losses to follow-up fully described and accounted for? Q8, Were adverse events or complications adequately documented? Q9, Were statistical analyses appropriate and clearly presented? Q10, Were conclusions consistent with the study data and not over-extended? Newcastle–Ottawa Scale (NOS) for non-randomized studies:Numbers I-VIII, in heading signified: I, representativeness of the exposed cohort; II, Selection of the non-exposed cohort; III, Ascertainment of exposure IV, Demonstration that outcome of interest was not present at start of study V, comparability of cohorts on the basis of design or analysis; VI, assessment of outcome; VII, Adequate follow-up length for outcomes to occur; VIII, Adequacy of follow-up of cohorts.

### Tumor response

**FIGURE 2:**
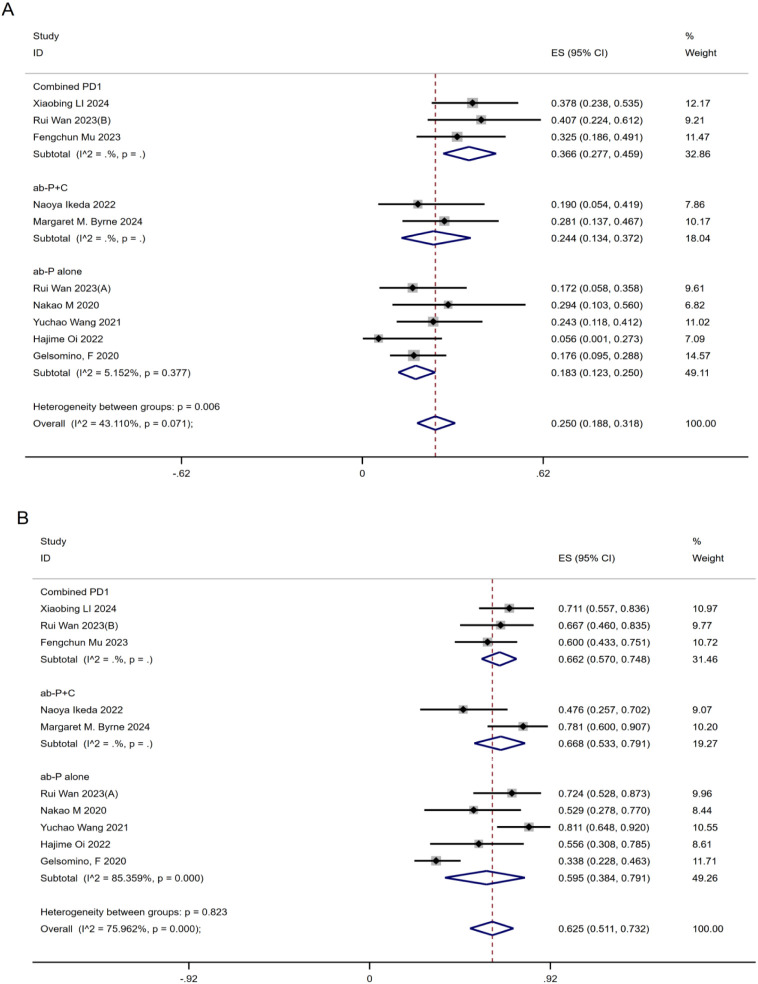
Forest plot about the pooled results of ORR **(A)** and DCR **(B)** in total by the treatment regimen subgroup. ORR, overall response rate; DCR, disease control rate.

All studies included in the analysis reported the efficacy response to nab-paclitaxel in the treatment of advanced or recurrent small cell lung cancer. The ORRs across the studies varied from 19% to 24%. The random-effects model was used because of significant heterogeneity (I^2^ = 43.11%, p = 0.071). The analysis showed a pooled ORR of 25.0% (95% CI: 18.8%–31.8%), and the ORR was further analyzed according to different nab-paclitaxel treatment regimens. Subgroup analysis revealed that the pooled ORR in patients who received nab-paclitaxel combined with PD-1 inhibitor was 36.6% (95% CI: 27.7%–45.9%), and nab-paclitaxel combined chemotherapy was 24.4% (95% CI: 13.4%–37.2%), Otherwise, the ORR of patients in nab-paclitaxel monotherapy was 18.3% (95% CI:12.3%–25.0%) (Figure 2A). All studies also included available data on DCR, and the pooled DCR was 62.5% (95% CI: 51.1%–73.2%), with significant heterogeneity (I^2^ = 75.962%, p = 0.000). Subgroup analysis showed that the pooled DCR in patients who received nab-paclitaxel combined with PD-1 inhibitor was 66.2% (95% CI: 57.0%–74.8%), and nab-paclitaxel combined chemotherapy was 66.2% (95% CI: 53.3%–79.1%). Otherwise, the patients in nab-paclitaxel monotherapy achieved a lower pooled DCR of 59.5% (95% CI: 38.4%–79.1%) (Figure 2B).

### Survival

**FIGURE 3:**
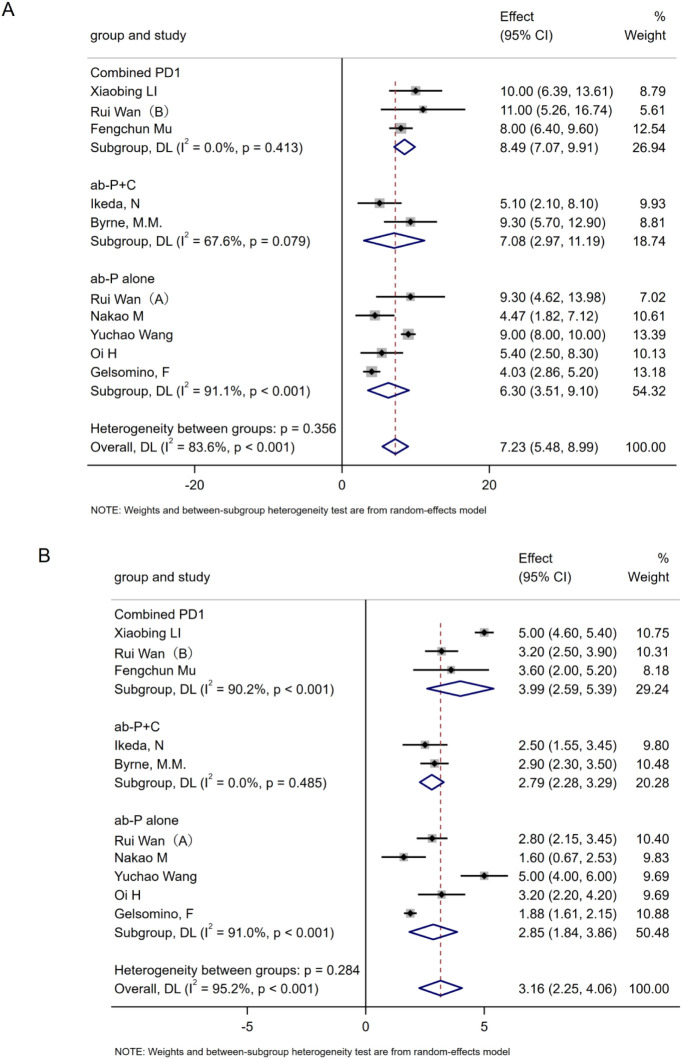
Forest plot about the pooled results of OS **(A)** and PFS **(B)** in total by the treatment regimen. OS, overall survival; PFS, progression-free survival.

All studies included in the analysis reported OS and progression-free survival PFS of all patients following nab-paclitaxel administration. In the random-effects model (I^2^ = 83.6%, p < 0.001), the pooled median OS was 7.23 months (95% CI 5.48–8.99 months). Subgroup analysis revealed that the pooled OS in patients who received nab-paclitaxel combined with PD-1 inhibitor was 8.49 months (95% CI 7.07–9.91 months), and nab-paclitaxel combined chemotherapy was 7.08 months (95% CI 2.97–11.19 months), Otherwise, the OS of patients in nab-paclitaxel monotherapy was 6.3 months (95% CI 3.51–9.10 months) as shown in Figure 3A. With regard to PFS, the random-effects model was performed (I^2^ = 95.2%, p < 0.001), and the results showed that the pooled median PFS was 3.16 months (95% CI:2.25–4.06 months), Subgroup analysis revealed that the pooled OS in patients who received nab-paclitaxel combined with PD-1 inhibitor was 3.99 months (95% CI 2.59–5.39 months), and nab-paclitaxel combined chemotherapy was 2.79 months (95% CI 2.28–3.29 months), Otherwise, the PFS of patients in nab-paclitaxel monotherapy was 2.85 months (95% CI 1.84–3.86 months) as shown in (Figure 3B).

### Toxicities

The most common adverse events (AEs), including all grades and grade ≥ III, associated with nab-paclitaxel in advanced or recurrent small-cell lung cancer (SCLC) were analyzed (Table 3). Most patients experienced grades 1–2 AEs, which were well tolerated. The results also indicated the three most commonly reported adverse events, including Leukopenia, Neutropenia, Anemia, with an incidence of 59.3% (95%CI:39.6%–77.6%), 54.9% (95%CI: 34.8%–74.3%), and 40.2% (95%CI: 19.7%–62.5%), respectively. Then, the most common non-hematologic toxicities included Fatigue (51.7%, 95%CI:31.0%–72.2%), gastrointestinal (37.0%, 95%CI:20.2%–55.6%), and Peripheral neuropathy (22.8%, 95%CI: 8.3%–41.3%). Additionally, the incidence rates of Liver toxicity AEs, reached 16.1% (95% CI: 3.8%–33.7%). The incidence of grade ≥ III adverse events were relatively rare, the most common is hematologic toxicities, including Neutropenia14.7% (95%CI:7.2%–23.9%) and Leukopenia14.1%, (95%CI:7.2%–22.6%), respectively.

**TABLE 3:** Adverse events of the studies included in the meta-analysis.

AE	All grade		≥Grade III	​
	ES,% (95 CI)	I^2^,%	ES,% (95 CI)	I^2^,%
Leukopenia	59.3 (39.6–77.6)	91.98	14.1 (7.2-22.60)	72.53
Neutropenia	54.9 (34.8–74.3)	92.42	14.7 (7.2-23.90)	76.14
Anemia	53.0 (31.8–73.7)	93.30	6.2 (1.10-13.90)	79.40
Thrombocytopenia	40.2 (19.7–62.5)	93.83	6.2 (0.80-15.0)	83.52
Fatigue	51.7 (31.0–72.2)	93.53	2.8 (0.20-7.30)	65.19
gastrointestinal	37.0 (20.2–55.6)	91.00	0.4 (0.00-1.80)	0.00
Peripheral neuropathy	22.8 (8.3–41.3)	91.92	0.0 (0.00-1.10)	0.00
Liver toxicity	16.1 (3.8–33.7)	92.43	0.6 (0.0-2.10)	0.00

### Sensitivity analysis

Sensitivity analysis was performed using a leave-one-out approach to assess the robustness of the pooled effect estimate. The overall pooled effect was 7.23 (95% CI: 5.48–8.99). Sequential exclusion of each individual study resulted in recalculated pooled effect sizes ranging from 6.97 to 7.48, all of which were highly consistent with the overall result. All 95% confidence intervals after exclusion overlapped substantially with the original interval, with no significant shift in effect direction. These findings indicate that the pooled result was highly stable and not driven by any single study, confirming the reliability of the meta-analysis conclusions. The results of sensitivity analysis are shown in Supplementary Figure 4.

**FIGURE 4:**
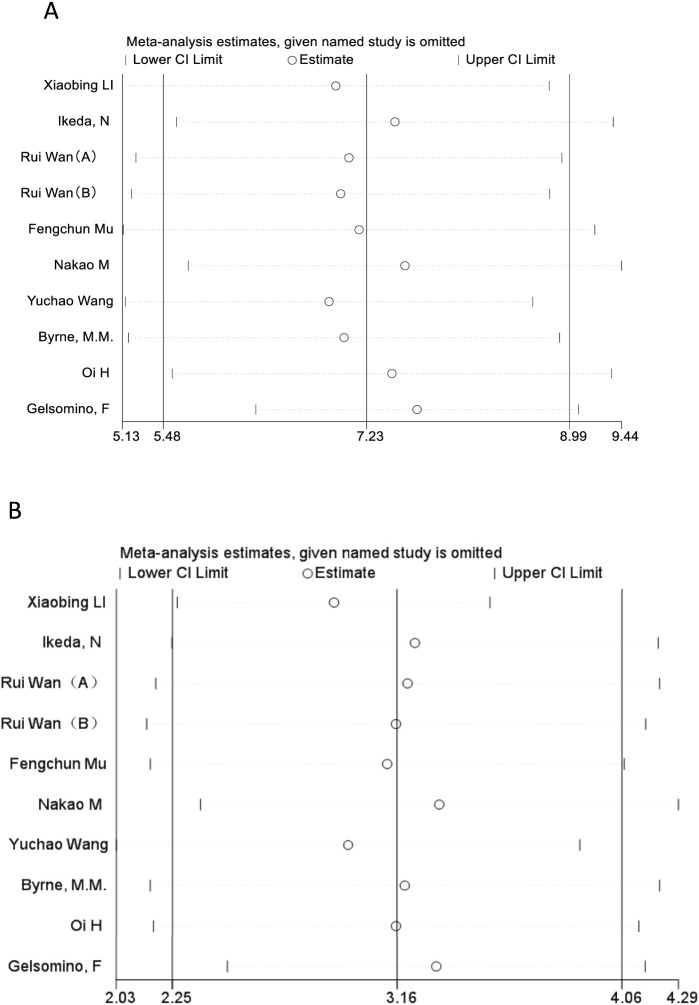
Sensitivity analysis results of OS **(A)** and PFS **(B)** in total by the treatment regimen. OS, overall survival; PFS, progression-free survival.

### Publication bias

The underlying logic of traditional publication bias tools (such as Egger’s regression and funnel plot asymmetry tests) relies on effect sizes having clear “positive” or “negative” directions. In a meta-analysis of proportions, a clear clinical definition of a “positive result” is lacking. More critically, the variance of single-arm proportion data is mathematically strictly bound to the magnitude of the proportion; as the effect size approaches 0 or 1, the variance naturally decreases, which can induce artificial asymmetry in proportion funnel plots and mislead bias judgments. The use of leave-one-out sensitivity analysis is an excellent alternative strategy (Sterne et al., 2011; Hunter et al., 2014). Given the inclusion of single-arm studies, formal assessment of publication bias using funnel plots and Egger’s test was not performed, as these statistical methods are not valid for non-comparative study designs. Instead, the robustness of the results was confirmed by sensitivity analysis.

## Discussion

This single-arm meta-analysis systematically included 9 studies involving 334 patients with advanced or recurrent small cell lung cancer (SCLC), aiming to clarify the efficacy and safety of nab-paclitaxel in later-line treatment, explore the optimal treatment strategy through subgroup analysis, and provide evidence-based basis for clinical decision-making. Combining the results of this study and the current progress in clinical research, the key findings, potential mechanisms, study limitations, and clinical significance are discussed as follows.

The results of this study showed that when nab-paclitaxel was used in the later-line treatment of advanced or recurrent SCLC, the pooled objective response rate (ORR) was 25.0% and the disease control rate (DCR) was 62.5%, indicating that this drug can effectively control tumor progression, relieve tumor burden, and provide certain clinical benefits for later-line patients. In terms of survival data, the pooled progression-free survival (PFS) was 3.16 months and the overall survival (OS) was 7.23 months. Subgroup analysis showed that the immunotherapy combination group was superior to other groups in ORR, PFS and OS. This result is similar to the overall data of standard second-line treatments recommended by most current guidelines, and shows no significant advantage in terms of overall efficacy.

Regarding safety, the most common treatment-related adverse events of nab-paclitaxel were hematologic toxicities. The highest incidences were leukopenia (59.3% all-grade, 14.1% grade ≥3), neutropenia (54.9% all-grade, 14.7% grade ≥3), and anemia (53.0% all-grade, 6.2% grade ≥3). However, the incidence of grade ≥3 severe hematologic toxicity was below 15%, and no treatment-related deaths were reported. Non-hematologic toxicities mainly manifested as fatigue, gastrointestinal reactions, and peripheral neurotoxicity, most of which were grade 1–2 and could be alleviated by symptomatic management without treatment interruption. These findings indicate that nab-paclitaxel has a manageable safety profile and favorable tolerability in later-line treatment. Compared with conventional later-line chemotherapeutic agents (such as topotecan) and solvent-based paclitaxel, its advantages of no requirement for steroid premedication, low hypersensitivity risk, and relatively mild hematologic toxicity further improve the acceptability of its clinical application. These findings indicate that nab-paclitaxel has a manageable safety profile and favorable tolerability in later-line treatment. Compared with conventional later-line chemotherapeutic agents (such as topotecan) and solvent-based paclitaxel, its advantages of no requirement for steroid premedication, low hypersensitivity risk, and relatively mild hematologic toxicity further improve the acceptability of its clinical application (Riemsma et al., 2010).

Subgroup analysis represents an important highlight of this study. The results demonstrated that combination therapy with immunotherapy was associated with numerically higher response rates and survival. Compared with previous immunotherapy monotherapy in later-line settings, the objective response rate (ORR) was improved. However, no significant advantage was observed in terms of overall progression-free survival (PFS) and overall survival (OS) (Chung et al., 2020; Ready et al., 2019; Ready et al., 2020). Similarly, in a phase II multicenter study, patients with SCLC progressing after first-line chemotherapy received paclitaxel plus pembrolizumab as second-line treatment, achieving an ORR of 23.1%, median PFS of 5.0 months, and median OS of 9.1 months, which were consistent with the findings of our study (Chung et al., 2020). This observation aligns with the synergistic mechanism of “chemotherapy plus immunotherapy” in recent cancer therapy. Immunotherapy can activate the host immune system and enhance the immunogenicity of tumor cells. Meanwhile, nab-paclitaxel not only directly kills tumor cells but also boosts the efficacy of immunotherapy by releasing tumor-associated antigens and remodeling the tumor microenvironment, thereby generating synergistic antitumor effects (Schmid et al., 2018; Soliman, 2017; Yang et al., 2026). These subgroup findings provide a novel optimized direction for later-line treatment of advanced or relapsed SCLC, suggesting that combination immunotherapy may serve as a preferred strategy for nab-paclitaxel in this clinical setting.

In recent years, many single-arm studies have explored the value of nab-paclitaxel in the later-line treatment of advanced or recurrent SCLC. However, the results have been somewhat inconsistent due to small sample sizes, substantial heterogeneity in first-line regimens, variable later-line strategies including combination agents and dosages, as well as differences in baseline patient characteristics and follow-up durations. For instance, one study reported an objective response rate (ORR) of 38% with nab-paclitaxel monotherapy in the second-line setting, which was higher than the 25.0% observed in the present study. This discrepancy may be attributed to differences in treatment regimens and lower tumor burden among enrolled patients in that study (Li et al., 2024). Another study reported a median progression-free survival (PFS) of 1.6 months, which was shorter than the 3.16 months in our analysis. This is presumed to be related to variations in follow-up methods, small sample size, and differences in baseline patient status, such as Eastern Cooperative Oncology Group performance status and the number of metastatic sites (Nakao et al., 2020).

By pooling data from 9 studies via meta-analysis, this study effectively reduced bias from single-center, small-sample investigations and improved the reliability of the results. Given that most studies in this field are currently designed as single-arm trials, our analysis focused on this study design to avoid heterogeneity caused by different control regimens, allowing a more focused evaluation of the efficacy and safety of nab-paclitaxel itself. Considering the relatively high heterogeneity in the overall pooled outcomes, we further performed subgroup analyses according to specific administration regimens, including subgroups of immunotherapy combination, monotherapy, and chemotherapy combination. Among them, the immunotherapy combination subgroup supplemented evidence-based support for nab-paclitaxel combination strategies, which is consistent with current trends in clinical practice (Horn et al., 2018; Paz-Ares et al., 2019).

Of note, the survival outcomes of nab-paclitaxel in this study (median PFS 3.16 months, median OS 7.23 months) remain inferior to those of first-line chemotherapy regimens such as etoposide plus platinum. This is closely associated with factors including rapid tumor progression, reduced physical tolerance, and increased drug resistance in patients receiving later-line therapy (Li et al., 2025; Zhou et al., 2021). It also reflects the severe challenges of later-line treatment for advanced or relapsed SCLC: curative therapies are still unavailable, and the therapeutic goals remain mainly to control symptoms, prolong survival, and improve quality of life. Given its efficacy and safety profile, nab-paclitaxel represents a reasonable option for such patients.

As a novel formulation of paclitaxel, nab-paclitaxel achieves targeted delivery to tumor tissues via an albumin-based nanoparticle carrier. Its mechanism of action mainly includes two aspects: On the one hand, albumin binds to the gp60 receptor on the surface of tumor cells and delivers paclitaxel into tumor cells through endocytosis. This increases local drug concentration in tumors, enhances antitumor activity, reduces drug exposure in peripheral normal tissues, and lowers the incidence of adverse reactions (Desai et al., 2006). On the other hand, paclitaxel exerts its tumoricidal effect by inhibiting microtubule polymerization in tumor cells, blocking cell division, and inducing tumor cell apoptosis (Yardley, 2013).

The synergistic mechanism of nab-paclitaxel combined with immunotherapy is currently recognized to involve three aspects. First, by killing tumor cells, nab-paclitaxel induces immunogenic cell death (ICD) and the release of tumor-associated antigens (TAAs). These antigens are captured by antigen-presenting cells (APCs) and naive T cells, thereby enhancing T cell-mediated tumor recognition and cytotoxicity (Chen et al., 2021; Vennin et al., 2023). Second, paclitaxel suppresses the activity of regulatory T cells (Tregs) and myeloid-derived suppressor cells (MDSCs) in the tumor microenvironment, reduces the secretion of immunosuppressive factors, and remodels the immune-suppressive tumor microenvironment (Chen et al., 2021; Vennin et al., 2023). Third, immunotherapy (such as PD-1/PD-L1 inhibitors) reverses immune escape of tumor cells and exerts synergistic antitumor effects with nab-paclitaxel, further enhancing therapeutic efficacy (Soliman, 2017; Chen et al., 2021). This synergistic mechanism also provides theoretical support for the subgroup finding that combination immunotherapy is superior to monotherapy and conventional chemotherapy.

Although this study summarized multi-center data through meta-analysis and improved the reliability of the results, there are still certain limitations that need to be considered in clinical interpretation. First, all studies included in this study are single-arm non-randomized controlled trials, lacking concurrent controls (such as direct comparison with traditional later-line chemotherapy drugs such as topotecan and irinotecan), so it is impossible to clarify the advantages of nab-paclitaxel compared with other later-line treatment regimens, and there may be selection bias (Tang et al., 2010); second, the sample size of the included studies is still relatively small (a total of 334 cases), and there is heterogeneity among the studies in terms of first-line treatment regimens, dosage and cycle of nab-paclitaxel, and follow-up cycle, which may affect the stability of the pooled results (IntHout et al., 2015); third, this study did not conduct further subgroup analysis on the baseline characteristics of patients (such as age, performance status score, metastatic sites, and previous treatment lines), so it is impossible to clarify the efficacy differences in patients with different baseline characteristics, which limits the precise application of the study results; finally, although this study has subdivided adverse reactions and clarified the specific types and incidence of non-hematological toxicities, it did not conduct subgroup analysis related to adverse reactions for different medication modes such as nab-paclitaxel monotherapy and combination therapy, so it was impossible to clarify the impact of different medication modes on the risk and type of adverse reactions, which may lead to an incomplete assessment of the safety of nab-paclitaxel.

The clinical significance of this study is mainly reflected in two aspects: first, by systematically quantifying the efficacy and safety of nab-paclitaxel, it clarifies its clinical value in the later-line treatment of advanced or recurrent SCLC, and provides a reliable evidence-based basis for clinicians to choose later-line treatment regimens. Especially for patients who cannot tolerate the adverse reactions of traditional chemotherapy or fail first-line treatment, nab-paclitaxel can be used as a safe and effective treatment option; second, subgroup analysis suggests that nab-paclitaxel combined with immunotherapy can further improve the efficacy, providing a new optimization direction for the later-line treatment of advanced or recurrent SCLC, which is expected to improve the survival prognosis of later-line patients.

From a methodological perspective, most studies included in this meta-analysis are single-arm non-controlled trials without concurrent internal control groups. The subgroup comparisons performed in this study are essentially indirect cross-trial comparisons, rather than head-to-head randomized controlled comparisons. Such unplanned indirect comparisons are inherently prone to multiple confounding biases, including differences across independent studies in patient baseline characteristics, disease stage composition, lines of prior treatment, follow-up duration, and inconsistent eligibility criteria. Statistical heterogeneity between subgroups only reflects inter-group variation in pooled effect sizes and cannot confirm causal relationships or definite clinical superiority among different treatment regimens. Accordingly, subgroup differences should not be overinterpreted as direct efficacy advantages of different therapeutic strategies.

In view of the limitations of this study, further research can be carried out in the following aspects in the future: first, conduct large-sample, multi-center, randomized controlled trials to directly compare nab-paclitaxel monotherapy or combined with immunotherapy with traditional later-line chemotherapy drugs, and clarify its efficacy advantages and applicable population; second, expand the sample size, include more patients with different baseline characteristics, conduct stratified subgroup analysis, explore the impact of factors such as age, performance status, and metastatic sites on efficacy, and achieve precise treatment; third, extend the follow-up period to observe the long-term efficacy and safety of nab-paclitaxel, especially the long-term survival benefit of combined immunotherapy; fourth, further explore the synergistic mechanism of nab-paclitaxel and immunotherapy to provide theoretical support for optimizing the combined treatment regimen and improving the treatment effect.

In addition, with the development of tumor precision medicine, gene detection technology can be combined in the future to screen out patient subgroups sensitive to nab-paclitaxel or combined immunotherapy, further improving the pertinence and effectiveness of treatment, and providing better treatment options for patients with advanced or recurrent SCLC. Given the inherent limitations of single biomarkers such as programmed death-ligand 1 (PD-L1), the design of future clinical trials should actively integrate emerging composite biomarker systems, including spatial analysis of the tumor immune microenvironment, characteristics of antigen presentation mechanisms, and dynamic monitoring of circulating tumor DNA (ctDNA). This strategy can optimize patient stratification and guide precision medication in clinical practice (Yang et al., 2026).

## Conclusion

In summary, our meta-analysis demonstrates the efficacy and safety of nab-paclitaxel in patients with advanced or recurrent small cell cancer, providing evidence for its future clinical application. However, since there are limited clinical data, future largescale and multiple-center RCTs are required to confirm this conclusion.

## Data Availability

The original contributions presented in the study are included in the article/supplementary material, further inquiries can be directed to the corresponding author.
